# Recurrent Endobronchial Coil Migration Associated With Needle Biopsy Following Pulmonary Artery Pseudoaneurysm Embolization

**DOI:** 10.1002/rcr2.70198

**Published:** 2025-04-29

**Authors:** Michael H. Koenig, Mina S. Makary, Tejas Sinha, Jeffrey C. Horowitz, Nicholas Pastis

**Affiliations:** ^1^ Division of Pulmonary, Critical Care, and Sleep Medicine, Department of Internal Medicine, Davis Heart and Lung Institute, College of Medicine The Ohio State University Wexner Medical Center Columbus Ohio USA; ^2^ Department of Radiology, College of Medicine The Ohio State University Wexner Medical Center Columbus Ohio USA; ^3^ OhioHealth Physician Group Interventional Pulmonology Columbus Ohio USA

**Keywords:** bronchoscopy, endobronchial coil migration, haemoptysis, pulmonary artery embolization, pulmonary artery pseudoaneurysm

## Abstract

Endobronchial coil migration is a rare complication of pulmonary artery embolization, occurring when coils erode through a vessel into an airway. We describe a patient who underwent embolization of pulmonary artery pseudoaneurysms associated with a lung mass. The unique aspect of this case is the development of recurrent endobronchial coil migration following a CT‐guided core needle biopsy and a subsequent endobronchial ultrasound‐guided transbronchial needle aspiration (EBUS‐TBNA) of the mass. In both circumstances, the portion of the coil that migrated into the large airways was cut with endoscopic scissors and removed with a flexible bronchoscope. This case highlights that while cutting and removing a portion of a migrated coil bronchoscopically is an effective approach, it can be associated with a risk of recurrent coil migration, haemoptysis, and pneumonia.

## Graphical abstract

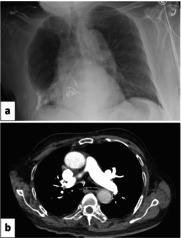

## 1 Introduction

Endobronchial coil migration is a rare complication of pulmonary artery embolization [1]. It occurs when coils erode through a vessel into an airway and has been described after embolization of pulmonary artery aneurysms [2, 3, 4, 5, 6], pulmonary arteriovenous malformations [7, 8, 9, 10, 11, 12], pulmonary artery pseudoaneurysms (PAPs) [1, 13, 14, 15], and bronchial arteries [16]. We describe a patient who developed endobronchial coil migration 8 weeks after successful endovascular embolization of two bleeding PAPs associated with a lung mass. Migration occurred following CT‐guided core needle biopsy of the mass and recurred after a subsequent endobronchial ultrasound‐guided transbronchial needle aspiration (EBUS‐TBNA). In both circumstances, the portion of the coil in the large airways was cut with endoscopic scissors and removed with a flexible bronchoscope. This is the first description of recurrent coil migration associated with needle biopsy of a lung mass. This case also highlights the potential risks of clipping and removing a portion of the coil bronchoscopically.

## 2 Case Report

**FIGURE 1:**
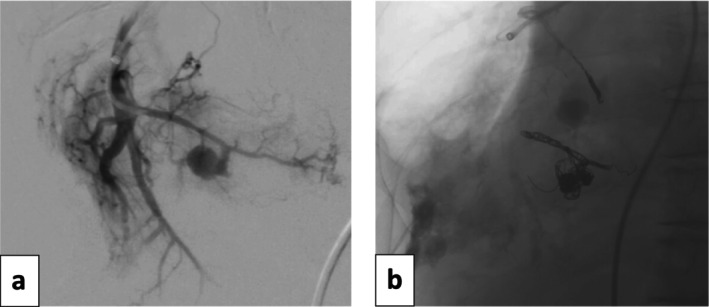
(a) Right pulmonary artery diagnostic angiography demonstrated two lower lobe bleeding pseudoaneurysms, which were successfully treated with superselective embolization (b). Coils were platinum with nylon and PGLA filaments added; Concerto, Medtronic Inc.; Dublin, Ireland. Sizes of the coils were 6 mm × 20 cm, 12 mm × 30 cm, 12 mm × 30 cm, 10 mm × 30 cm, 7 mm × 30 cm, and 5 mm × 20 cm.

An 88‐year‐old man was admitted to the hospital with massive haemoptysis. Past medical history was notable for a 6.8 × 7.2 cm right lower lobe mass, distant tuberculosis infection, recent pulmonary embolism, and a 30 pack‐year smoking history (quit 20 years ago). CT angiography demonstrated two subcentimetre‐enhancing foci within the necrotic right lower lobe mass, and conventional angiography of the right pulmonary artery confirmed bleeding pseudoaneurysms (Figure 1a). Two separate third‐order branches of the right lower lobe pulmonary artery and associated pseudoaneurysms were subsequently embolized using microcoils (platinum coils with nylon and PGLA filaments added; Concerto, Medtronic Inc.; Dublin, Ireland) with resolution of haemoptysis (Figure 1b).

**FIGURE 2:**
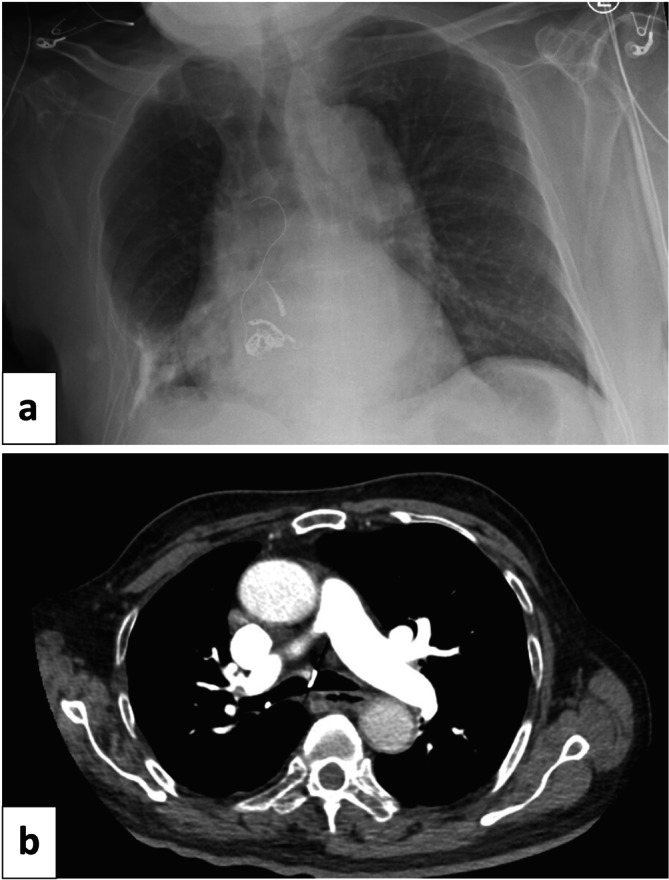
(a) Chest x‐ray demonstrated partial unwinding of one of the embolization coils. (b) Chest CT confirmed endobronchial coil migration to the level of the carina.

**FIGURE 3:**
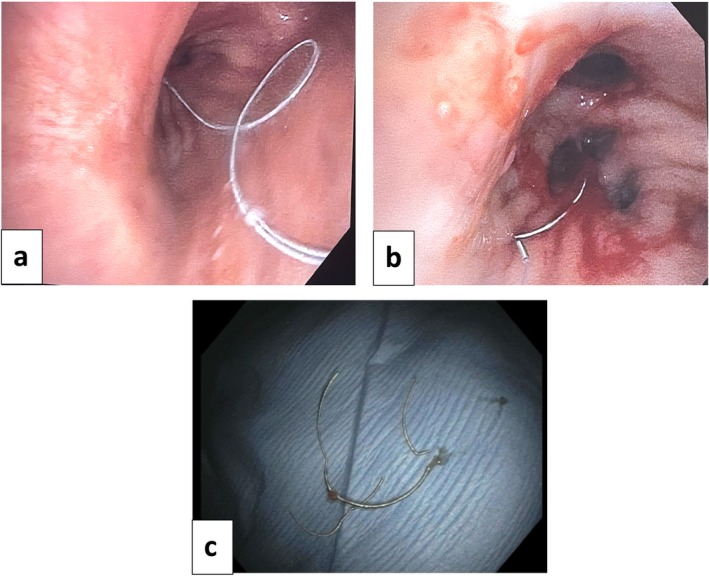
(a) Image of right mainstem bronchus during initial bronchoscopy demonstrated coil originating from the right lower lobe and terminating at the level of the carina. (b) Small portion of coil remained in the right lower lobe after clipping with endoscopic scissors and removing from larger airways with forceps through a flexible bronchoscope. (c) Coil removed during bronchoscopy.

Eight weeks after this coil embolization, a CT‐guided core needle biopsy of the mass was performed. Six passes were completed with a 20‐gauge coaxial biopsy device of the inferior‐medial portion of the mass. There were no complications during the procedure. The biopsy revealed atypical glandular proliferation within a fibroinflammatory background. Bacterial, fungal, and AFB smears and cultures were negative. The patient developed intractable coughing, and a chest radiograph 1 h following the biopsy demonstrated unfurling of the coil into the airway with extension into the trachea (Figure 2a). Chest CT confirmed endobronchial migration of the coil with the distal tip at the level of the carina (Figure 2b). Due to the risk of recurrent bleeding, instead of removing the entire coil, endoscopic scissors were used to cut sections of the coil, which were removed with forceps through a flexible bronchoscope (Figure 3a,c). At the conclusion of the procedure, only a small portion of the coil remained visible in the right lower lobe bronchus (Figure 3b).

**FIGURE 4:**
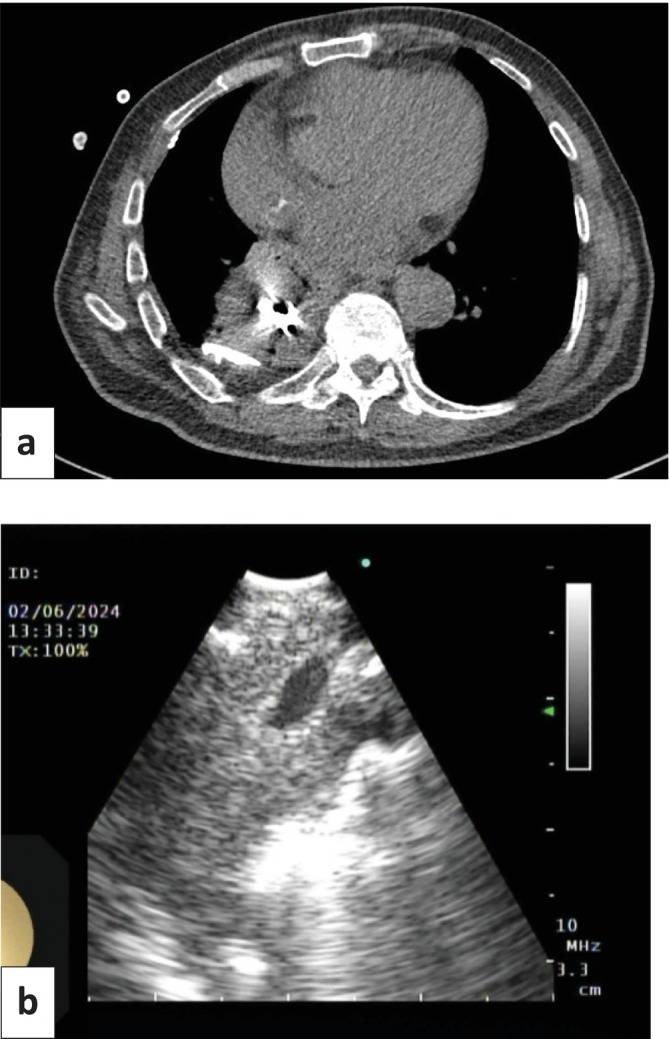
(a) Chest CT redemonstrated 6.8 × 7.2 cm right lower lobe mass with embolization coils in the centre, stable in size compared to 6 months prior. Pleural calcifications and a trace right pleural effusion were also noted. (b) Endobronchial ultrasound image of right lower lobe mass, notable for a vessel coursing through the mass as well as embolization coils, which were avoided during biopsy.

Chest CT 6 months later redemonstrated the lung mass, which was relatively stable in size (Figure 4a). Because of continued suspicion for malignancy, a repeat biopsy was pursued. The patient underwent EBUS‐TBNA because coil packing prevented access to the central portion of the mass via percutaneous biopsy. Coils and a vessel within the mass were visualised with ultrasound and avoided during the biopsy (Figure 4b). Seven total passes of the right lower lobe mass were performed with an Olympus Vizi Shot 1 (21‐gauge) needle. No coil was seen in the airways. There were no complications. Biopsies demonstrated collections of neutrophils and submucosal lymphoplasmacytic inflammation. Bacterial, fungal, and AFB smears and cultures were again negative. Given the lack of a definitive diagnosis, the patient was discussed at a multidisciplinary tumour board. It was determined that he was not a candidate for surgical biopsy or resection of the mass. Stereotactic body radiation therapy was discussed but not offered because of the absence of a definitive cancer diagnosis.

Ten weeks following EBUS‐TBNA, the patient presented with pneumonia and recurrent haemoptysis. Repeat bronchoscopy identified purulent secretions in the right lower lobe with recurrent endobronchial migration of the coil to the level of the carina. Portions of the coil were again cut with endoscopic scissors and removed bronchoscopically, with subsequent resolution of haemoptysis. The pneumonia was treated with antibiotics. Interval chest CT demonstrated no coil in the airways and a decrease in the size of the mass. This improvement suggested that at least a component of the mass was secondary to chronic inflammation and/or infection, likely related to aspiration, which was reported clinically and seen on modified barium swallow.

The patient again presented to the hospital less than 4 months later with massive haemoptysis requiring emergent intubation and placement of a 7 Fr endobronchial blocker in the bronchus intermedius. No coil was seen in the airways during bronchoscopy, but there was active bleeding originating from the right lower lobe. The patient continued to have haemoptysis despite embolisation of the right intercostobronchial artery with Gelfoam, treatment with nebulised tranexamic acid, and placement of an endobronchial valve in the right lower lobe bronchus. No further bronchoscopic or endovascular treatment options remained, and the patient was not a surgical candidate. The patient's family decided to transition to comfort focused care, and he died after an 18‐day hospitalisation.

## 3 Discussion

Pulmonary artery pseudoaneurysms (PAPs) are uncommon and defined as the focal dilation of a segment of a pulmonary artery involving only the external layers of the arterial wall [17]. They can either be congenital or acquired from infection, neoplasm, trauma, or vasculitis [17]. If left untreated, mortality is high secondary to rupture [17]. Endovascular interventions like coil embolization have surpassed surgical management of PAPs as the treatment of choice [18]. While pulmonary artery embolization has a low rate of complications, risks include recanalization, coil migration, misplacement of coils, damage to the vessel wall, and delayed bacterial contamination [13]. Of these complications, endobronchial coil migration occurs rarely. It was first described in 1990, developing when a coil erodes through the vessel and into an airway [2]. Lacking an intact vascular wall, PAPs may have an increased risk for coil penetration and migration [15]. Patients can be asymptomatic [2] or present with haemoptysis [3, 5, 6, 11, 12, 13, 14, 19], cough [4, 6, 7, 9, 13, 15, 16, 19], hoarseness [16], or expectoration of a coil [3, 4, 6, 9, 13, 16]. The duration of time from the initial embolization to coil migration is variable, ranging from weeks [10] to over two decades [11].

Endobronchial coil migration often occurs in the setting of inflammation from infection or neoplasm. This results in the development of friable tissue with impaired wound healing that is more susceptible to damage and the persistence of an otherwise transient arterial to bronchial fistula [1]. In three reported cases of endobronchial coil migration following PAP embolization, the contributing inflammation was attributed to tuberculosis [1], advanced squamous cell carcinoma [14, 15], and radiation therapy [14]. In the current case, the necrotic lung mass and associated inflammation likely increased the chance of a fistulous connection between the bronchus and pulmonary artery, resulting in endobronchial coil migration. This case is the first to describe recurrent endobronchial coil migration following CT‐guided core needle biopsy and a subsequent EBUS‐TBNA. The temporal relationship suggests that needle biopsy of the adjacent mass disrupted the coil and facilitated migration.

Management options of endobronchial coil migration include surgical intervention [2, 3, 5, 6, 9, 12, 13, 14], such as lobectomy or segmentectomy, bronchoscopic removal of all or portions of the coil [1, 4, 6, 7, 16, 19], and observation [11, 15]. A multidisciplinary discussion is often beneficial to assess the risks and benefits of each option and choose the safest and most effective approach for the patient [4, 19]. In the current case, after discussion amongst interventional radiology, interventional pulmonology, and thoracic surgery, it was determined that the risk of haemorrhage was too high with complete removal of the coil. Surgery was not a viable option due to the patient's age, comorbidities, and poor functional status. Previously described bronchoscopic strategies included cutting the coil with laparoscopic scissors [4], loop cutters [1, 16], or endobronchial scissors [1]. Forceps were then used to remove either the entire coil [6, 7, 19] or only the clipped pieces, with residual coil remaining in the distal airways [1, 4]. In one case, an endovascular plug was placed within the bronchus at the site of coil erosion to prevent further coil migration and haemorrhage [4]. Two cases documented use of rigid bronchoscopy [4, 19]. For our patient, flexible bronchoscopy and endoscopic scissors were utilised to clip pieces of coil. Forceps were then employed to remove these pieces from the large airways, with only a small portion remaining in the right lower lobe bronchus. Of the eight cases that utilised bronchoscopic management or observation, only one was notable for recurrence of haemoptysis and coil migration [6]. In that case, bronchoscopy was used to completely remove all visible coil from the airways during the initial presentation with subsequent resolution of haemoptysis. Four years later, the patient presented with cough and haemoptysis with evidence of recurrent coil migration to the trachea. This occurrence was successfully managed with lobectomy. Our patient was followed over the course of a year after his initial presentation. The current case supports the efficacy of bronchoscopic management with cutting portions of the coil, particularly in the short‐term, as it led to cessation of haemoptysis and improvement in cough. However, it also highlights some of the possible complications with this approach, including recurrent coil migration, haemoptysis, and pneumonia.

In conclusion, this case illustrates an association between needle biopsy of a lung mass and subsequent endobronchial coil migration. If the patient is a poor surgical candidate or the risk of bleeding with complete removal is excessive, bronchoscopic cutting and removal of a portion of the migrated coil can be an effective approach. However, it is associated with a risk of recurrent coil migration, haemoptysis, and pneumonia.

## Author Contributions

Michael H. Koenig drafted the manuscript. All authors listed critically reviewed the manuscript and approved the version to be published.

## Ethics Statement

The authors declare that appropriate written informed consent was obtained for the publication of this manuscript and accompanying images.

## Conflicts of Interest

The authors declare no conflicts of interest.

## Data Availability

Data sharing is not applicable to this article as no new data were created or analyzed in this study.

## References

[1] J. N. McLaughlin , D. Lamus , S. Hegde , and S. P. Kalva , “Endobronchial Migration of a POD Packing Coil Following Embolization of a Pulmonary Artery Pseudoaneurysm,” Seminars in Interventional Radiology 40, no. 5 (2023): 407–410, 10.1055/s-0043-1774408. 37927515 PMC10622237

[2] J. Abad , R. Villar , G. Parga , et al., “Bronchial Migration of Pulmonary Arterial Coil,” Cardiovascular and Interventional Radiology 13, no. 6 (1990): 345–346, 10.1007/BF02578671. 2126989

[3] M. Elhusseiny , A. Moawad , D. AbdAlla , and T. Amer , “Pulmonary Artery Coil: Unexpected Expectorated Foreign Body,” Chest 149 (2016): A421.

[4] J. M. Crawford , P. P. Patel , A. R. DuCoffe , M. Tsai , and J. A. Hodgson , “Endovascular Plug for Endobronchial Management of an Expectorated Pulmonary Artery Embolization Coil: A Case Report,” Annals of Allergy, Asthma & Immunology 17, no. 2 (2023): e01663, 10.1213/XAA.0000000000001663. 36779890

[5] S. Y. Park , M. J. Chung , K. J. Chae , H. B. Lee , and S. J. Park , “Bronchial Impaction of Arterial Coil,” American Journal of Respiratory and Critical Care Medicine 197, no. 11 (2018): 1481–1482, 10.1164/rccm.201708-1582IM. 29782807

[6] D. M. Hansen, Jr. and C. Dyke , “Pulmonary Artery‐Tracheal Fistula After Coil Implantation for Behcet's Disease,” Cureus 12, no. 10 (2020): e11036, 10.7759/cureus.11036. 33214963 PMC7673279

[7] T. X. Hu , S. S. Oh , and J. P. McWilliams , “Bronchoscopy‐Guided Removal of Intrabronchial Coil Migration After Coil Embolization of Pulmonary Arteriovenous Malformation,” Radiology Case Reports 17, no. 9 (2022): 3410–3414, 10.1016/j.radcr.2022.06.078. 35899085 PMC9309579

[8] G. P. Loke , D. A. Story , F. Liskaser , and S. Seevanayagam , “Pulmonary Arteriovenous Malformation Causing Massive Haemoptysis and Complicated by Coronary Air Embolism,” Anaesthesia and Intensive Care 34, no. 1 (2006): 75–78, 10.1177/0310057X0603400105. 16494154

[9] A. Konno‐Yamamoto , S. Yamamoto , J. Suzuki , T. Fukami , M. Kitani , and H. Matsui , “Migrated Coil Expectorated 12 Years After Embolization of Pulmonary Arteriovenous Malformation, due Probably to Abscess Formation Around the Coil,” Respiratory Medicine Case Reports 31 (2020): 101245, 10.1016/j.rmcr.2020.101245. 33083222 PMC7554207

[10] N. A. Yetkin and N. Tutar , “Intravascular Coil Migration to Bronchus: Review of the Literature With Two Case Reports,” Tüberküloz ve Toraks 67, no. 4 (2019): 307–313. English, 10.5578/tt.69010. 32050873

[11] C. So , M. Suzuki , Y. Iwaki , et al., “Unexpected Complications 25 Years After Coil Embolization for Pulmonary Arteriovenous Fistula,” Internal Medicine 62, no. 10 (2023): 1521–1525, 10.2169/internalmedicine.0560-22. 36198600 PMC10258102

[12] T. Umehara , M. Aoki , G. Kamimura , et al., “Coil Intrabronchial Migration in an Arteriovenous Malformation Patient Treated 10 Years Ago,” Annals of Thoracic and Cardiovascular Surgery 23, no. 4 (2017): 200–202, 10.5761/atcs.cr.16-00250. 28450683 PMC5569255

[13] A. M. Budacan , A. J. Patel , H. Foss , V. Abiuso , A. Ganeshan , and M. Kalkat , “Late Endovascular Coil Migration Following Traumatic Pulmonary Artery Pseudoaneurysm Embolization: Case Report,” Journal of Cardiothoracic Surgery 17, no. 1 (2022): 87, 10.1186/s13019-022-01841-7. 35477517 PMC9044845

[14] A. Schwertner , R. M. Kohlbrenner , E. J. Seeley , and R. P. Lokken , “Nonfibered Packing Coil Embolization of Pulmonary Artery Pseudoaneurysm Resulting in a Delayed Endobronchial Coil Migration,” Journal of Vascular and Interventional Radiology 32, no. 4 (2021): 626–628, 10.1016/j.jvir.2020.12.026. 33526345

[15] S. Elangovan and C. W. Too , “Embolisation of Large Pulmonary Artery Pseudoaneurysm With Conservative Treatment of Delayed Coil Extrusion,” Singapore Medical Journal 61, no. 3 (2020): 162–164, 10.11622/smedj.2020031. 32488273 PMC7905110

[16] H. Ishikawa , N. Omachi , M. Ryuge , J. Takafuji , and M. Hara , “Erratic Coil Migration in the Bronchus After Bronchial Artery Embolization,” Respirology Case Reports 7, no. 8 (2019): e00478, 10.1002/rcr2.478. 31463064 PMC6705188

[17] B. Guillaume , A. Vendrell , X. Stefanovic , F. Thony , and G. R. Ferretti , “Acquired Pulmonary Artery Pseudoaneurysms: A Pictorial Review,” British Journal of Radiology 90, no. 1073 (2017): 20160783, 10.1259/bjr.20160783. 28337922 PMC5602174

[18] A. Zugazaga , M. A. Stachno , A. García , et al., “Pulmonary Artery Pseudoaneurysms: Endovascular Management After Adequate Imaging Diagnosis,” European Radiology 31, no. 9 (2021): 6480–6488, 10.1007/s00330-021-07819-8. 33713173

[19] J. Doan , K. Puchhalapalli , P. J. Patel , M. Gasparri , J. S. Kurman , and B. S. Benn , “Bronchoscopic Extraction of a Migrated Endovascular Coil,” Thorax 77, no. 5 (2022): 526–527, 10.1136/thoraxjnl-2021-216873. 34353924

